# Application of high rate integrated anaerobic-aerobic/biogranular activated carbon sequencing batch reactor (IAnA-BioGACSBR) for treating strong municipal landfill leachate

**DOI:** 10.1038/s41598-017-02936-1

**Published:** 2017-06-08

**Authors:** Meghdad Pirsaheb, Hooshyar Hossini, Marius Sebastia Secula, Molouk Parvaneh, Ghulam Md Ashraf

**Affiliations:** 1 Department of Environmental Health Engineering, Faculty of Health, Kermanshah University of Medical Sciences, Kermanshah, Iran; 2 Technical University of Iași, Faculty of Chemical Engineering and Environmental Protection, Chemical Engineering Department, Iasi, Romania; 3 Student Research Committee, Kermanshah University of Medical Sciences, Kermanshah, Iran; 4 King Fahd Medical Research Center, King Abdulaziz University, Jeddah, Saudi Arabia

## Abstract

The aim of the present study is to evaluate the application of high rate integrated anaerobic-aerobic/biogranular activated carbon sequencing batch reactor (IAnA-BioGACSBR) to treat raw strong leachate from open dumping of municipal solid waste. The influence of two important and effective independent variables, COD concentrations and volumetric filling rate with GAC, onto the leachate treatment were investigated. Three responses such as TKN, BOD and COD were considered for evaluating the interaction of parameters. The results showed that maximum BOD_5_ removal of 98.9% in anaerobic zone and 99% in aerobic zone was obtained at the highest values of COD (~30000 mg/L) and filling ratio (~50%). The highest values of COD removal efficiency were found to be 98.54% and 98%, at COD rate of 10000 mg/L and GAC of 35%, respectively. The highest removal values of TKN was 77.2% and 78.9% in anaerobic and aerobic zone, respectively. Under optimal conditions, compared with the SBR and the GAC-SBR performances, results reveal that the application of the GAC-SBR has shown better effluent characteristics. Based on the results, it can be asserted that the application of the high rate IAnA-BioGACSBR for the treatment of biodegradable landfill leachate was more effective.

## Introduction

The special characteristics of municipal landfill leachate (MLL) including high concentrations of inorganic, organic, microbial, and chemical pollutants can potentially have hazardous and toxic effects on the ecosystem and environment^1^. Fresh MLL is of high strength due to low pH, high amounts of biochemical oxygen demand (BOD)_5_ and chemical oxygen demand (COD), and high toxic/hazardous contents^2^. Also, the young leachate may be up to 36 times stronger than raw sewage in regard of the COD content, but the mature leachate can be approximately similar^3, 4^. For biodegradable MLL, when the BOD_5_/COD ratio is higher than 0.3, biological techniques are used successfully for simultaneous removal of organic carbon and nitrogen^5^. For simultaneous treatment of the carbon and nitrogen content of MLL, many biological techniques such as ammonification, nitrification/denitrification and Anammox were investigated^6^. Due to the complex characteristics of MLL, for the confidence of final usage or discharge into the environment, it is occasionally necessary for MLL to be discharged into the municipal sewage system^7, 8^. Due to incompatibility of the conventional method with leachate properties, the landfill sites lack a system to treat their wastewater, and also MLL treatment is a difficult and expensive task^9^. To avoid the adverse impacts, high treatment costs and lower effectiveness of alternative treatment techniques, the biological methods have been used for MLL treatment^2, 8, 10^. Biological methods can be classified into aerobic, anaerobic and anoxic processes which can be used for treating biodegradable wastewaters, often characterized by a wide variation in composition^11^. These groups of treatment processes are environment friendly, reliable, simple and high cost-effective, and have been demonstrated historically to be very effective in removing the bulk of MLL with the high ratio of BOD_5_/COD^12^. Recently, among the biological methods, conventional activated sludge and sequencing batch reactors (SBRs), biofilters and moving bed processes have attracted major interests. On the other hand, these techniques proved to be quite effective in removing the organic matter and nutrients^2, 10^. SBRs are of high flexibility, simple to construct, cost-effective in installation and operation, highly efficient, have good settle ability, lower capital cost, and also have the ability for integration with aerobic and anaerobic processes^13, 14^. New studies and manipulations of SBR are still of high interest. Easy management and effectiveness under different operating conditions [hydraulic retention time (HRT) and organic loading rate] are other special properties that promote wide application of SBR^7^. It can be seen from the Table 1 that there are numerous operating possibilities for SBR and its combination with other processes for wastewater treatment. In recent years, various combined processes such as anaerobic and aerobic system, coagulation-flocculation, chemical and electrochemical oxidation have been considered for the enhanced treatment of leachate (see Table 1). For example, Aziz *et al*.^15^ have reported that simultaneous removal of ammonium and organic carbon from landfill leachate by anaerobic and aerobic systems is possible^15^. This process combination was supported by some advantages including simplicity, cost-effectiveness, high effluent quality, simultaneous organic and nitrogen removal, lower energy consumption and less sludge production^16^. In the combination of anaerobic-aerobic processes, though there is some biodegradability resistance, intensive degradation of MLL occurs. In addition, this coupled process can be used as pretreatment of strong wastewater such as MLL to tolerant levels.

**Table 1:** Various operations and combination of SBRs for wastewater treatment.

SBR type	Wastewater type	Objects	References
Coupling ASBR and modified SBR	Immature landfill leachate	Nitrogen and COD removal	Wang *et al*.^29^
UASB-SBR system	Landfill leachate	nitrogen	Sun *et al*.^30^
Electro-Fenton oxidation-SBR	Old aged landfill leachate	COD, BOD, SS, NH_3_-N, turbidity	Lin *et al*.^31^
Anaerobic–anoxic/nitrification sequencing batch reactor (A2N-SBR)	Domestic wastewater	Phosphorus and nitrogen removal	Wang *et al*.^32^
Aerobic Granules/SBR	Soybean-processing wastewater	formation of granule	Su *et al*.^33^
Anaerobic/Sequencing batch reactor (AnSBR)	Dairy wastewater	hydrogen (H_2_) production, COD removal	Venkata Mohan *et al*.^34^
Up-flow anaerobic sludge blanket/sequencing batch reactor (UASB-SBR)	Ammonium-rich landfill leachate	enhanced COD and TN removal	Sun *et al*.^16^
Granular anammox SBR	Urban landfill leachate	Nitrogen and COD removal	Ruscalleda *et al*.^35^
SBR-anammox	Urban landfill leachate	Nitrogen, TOC and COD removal	Ganigué *et al*.^36^
SBR-zeolite	Synthetic wastewater	nitrogen removal	Jung *et al*.^37^
A sequencing batch biofilm reactor (SBBR)	Synthetic wastewater	nitrogen removal	Yu *et al*.^38^

Activated carbon is a popular adsorbent due to its unique properties such as large porous surface area, thermo-stability and low acid/base reactivity, controllable pore-structure, ability to remove a wide range of pollutants and is can be used as polishing process following biological treatment^2, 17, 18^. Integration of activated carbon with a biological process can improve the treatment characteristics of the effluent due to highly available surface area and micro/macro pore for bacterial attachment and biofilm formation. On the other hand, resistant and recalcitrant pollutants can be absorbed in porous site and they are better placed in contact to biofilm and bacterial enzymes and therefore a better degradation is provided. Other advantages for the use of activated sludge together with biological processes include microorganisms protection (both autotrophic and heterotrophic) from organic loading shocks and prevention from bacteria washout, improvement in settleability and dewaterability of sludge, and the activated sludge bioregeneration^19^. However, the aim of present work is to evaluate the performance of a pilot-scale of the high rate IAnA-BioGACSBR for the treatment of strong MLL. The second aim is the optimization of the leachate treatment process in regard to COD and BOD criteria for discharging the treated effluent in the municipal wastewater collection network. Based on available database, no similar work has been reported so far. To analyze, model and optimize the significant responses [such as total COD, BOD and total kjeldahl nitrogen (TKN) removal] as a function of the two independent variables (COD concentrations and filling percent), full factorial design (FFD) and response surface methodology (RSM) were employed.

## Material and Methods

### Wastewater properties

Leachate samples were collected from the landfill site in Kermanshah, Iran. The samples were taken from landfill site and stored at 4 °C prior to use by minimizing the biological activity and chemical reactions. MLL characteristics are shown in Table 2.

**Table 2:** Characteristics of MLL.

Parameters	Unit	Maximum	Mean ± SD	Minimum
TCOD	mg/L	64000	38769.2 ± 12567.1	12800
sCOD	mg/L	46400	32886.1 ± 11404.1	11840
BOD_5_	mg/L	45000	27300 ± 2531.5	9600
NH_4_-N	mg/L	2886	2053 ± 832	1464
TKN	mg/L	3698. 8	2571.4 ± 474.7	2016
TP	mg/L	125.8	73.7 ± 43.8	24.3
Total suspended solids	mg/L	46847	19883 ± 10001.7	7000
Conductivity	mS/cm	93.7	71.2 ± 21.6	28.9
Turbidity	Nephelometric turbidity unit (NTU)	2910	1982.6 ± 526.3	1242
Alkalinity	mg/L as CaCO3	3200	1900.5 ± 567.7	950
pH	—	7.8	7.3 ± 0.3	6

### Reactor setup

**Figure 1:**
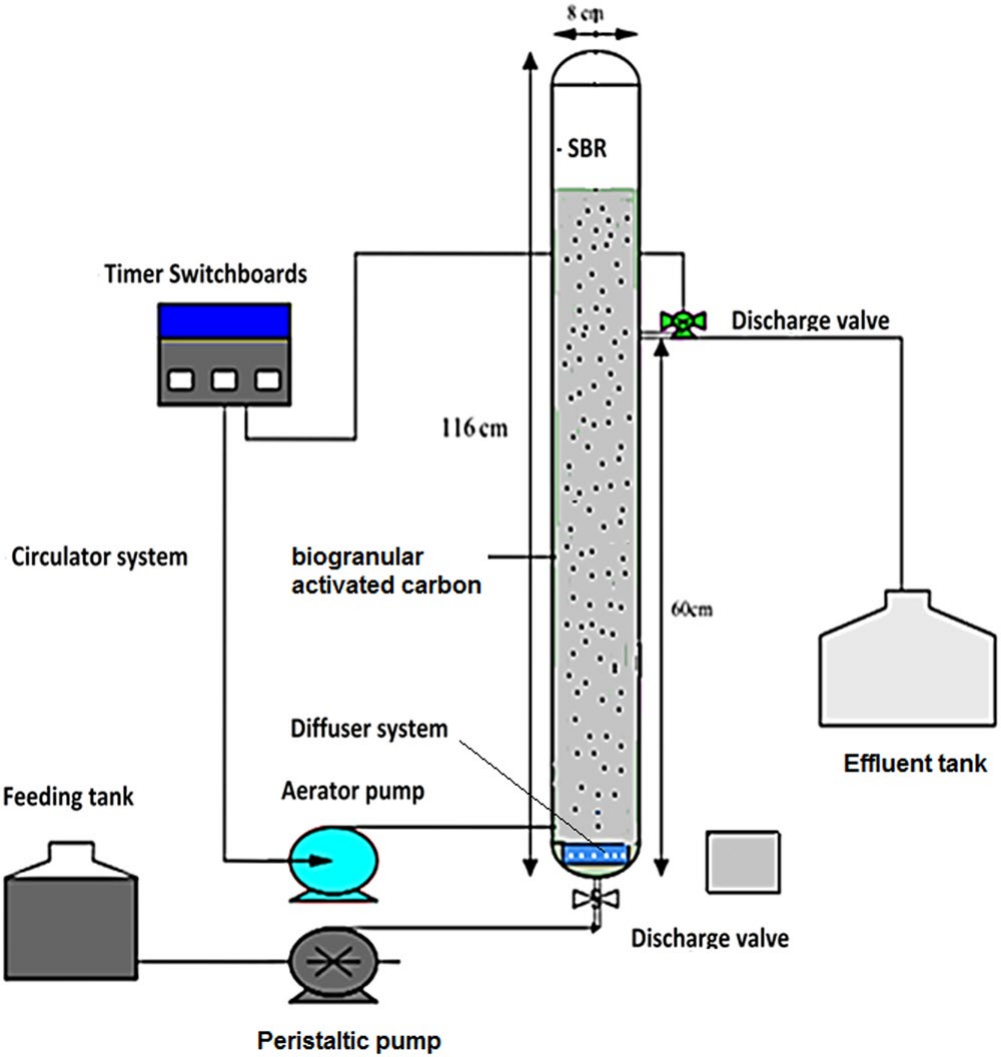
Schematic diagram of integrated anaerobic-aerobic/biogranular activated carbon sequencing batch reactor (IAnA-BioGACSBR).

A cylindrical Plexiglas reactor was used as SBR with a working volume of about 3.6 L (internal diameter ~6 cm and total height ~164 cm). The schematic plan of the combined system is illustrated in Fig. 1. By a preprogrammed timer (multi-Function, 4-OUT) the anaerobic and aerobic conditions were implemented. The reactor content was mixed using a circulator pump, and an aquarium pump (Hailea ACO-9602, China) was placed to supply air through a porous stone diffuser that was located at the bottom of the reactor. The reactor was covered with aluminum foil to prevent evaporation. The dissolved oxygen (DO) amount was maintained about more than 2.5 mg/L. Granular activated carbon (GAC) with a surface area about 900 m^2^/g and density 1.3 g/cm^3^ were added to SBR. To achieve the desirable loading rate (10000 < COD < 30000 mg/L), leachate was diluted with distillation water. The reactor has been inoculated with mixed culture of biomass obtained from the wastewater treatment plant of Farabi treatment plant, Kermanshah, Iran. The SBR was initially inoculated with 300 mL of mixed liquor, 20% volume with GAC (400 mL), and 1 L of its empty volume was loaded using diluted MLL and the initial concentration of COD was about 10000 mg/L. To prepare the excess bio-GAC for higher GAC filing ratio percentage of 35% and 50%, a parallel and similar condition was generated separately with IAnA-BioGACSBR and bioGAC. During the start-up period, HRTs in anaerobic as well as aerobic reactor were set equally at 12 h. To achieve high bacteria ability, the activated sludge was acclimatized for 45 days. The reactor was operated in following sequence; filling (5 min), anaerobic agitation (12 h), withdrawal (10 min), aeration (12 h), withdrawal (10 min) and settlement (1 h). In next step, to determine the optimum HRT for maximum COD removal, the system was operated at different anaerobic–aerobic contact times. This phase was performed as follows: anaerobic (24, 48 and 72 h) and aerobic (12, 24 and 36 h).

### Experimental design and mathematical modeling

The main part of the study was carried out to investigate the effect of the factors “GAC filling ratio” and “COD concentration”. The operating conditions are summarized in Table 3. FFD and RSM were used for the analysis of data and process optimization. FFD was established through Design Expert Software version 7. To optimize an analysis, independent effective variables with certain ranges including inlet COD concentration (*x*_*1*_) and volumetric filling rate (*x*_*2*_), were selected at three levels. The range and levels of the variables in coded and actual units are given in Table 4. The two operating variables were considered. COD, BOD and TKN removal were measured or calculated as per the response. The obtained experimental conditions and results are shown in Table 5. The results were analyzed by analysis of variance (ANOVA). Three-dimensional plots with the respective contour plots were obtained from the results of the experiments.

**Table 3:** Operational conditions.

Parameters	Range
DO (mg/L)	>2.5
HRT (anaerobic)(h)	12, 48, 72
HRT (aerobic)(h)	12, 24, 48
Designed MLSS (mg/L)	5000
COD (mg/L)	10000, 20000 and 30000
Volumetric filling rate with GAC (%)	20, 35 and 50

**Table 4:** Experimental range and levels for independent variables.

Variables	Symbol	Levels		
		−1	0	+1
COD (g/L)	*x*_*1*_	10	20	30
Filling rate with GAC (%)	*x*_*2*_	20	35	50

**Table 5:** Experimental conditions and results.

Run	Variables		Responses					
			COD removal, %		BOD removal, %		TKN removal, %	
	*x*_*1*_ (mg/L)	*x*_*2*_ (%)	Anaerobic zone	Aerobic zone	Anaerobic zone	Aerobic zone	Anaerobic zone	Aerobic zone
1	30000	35	97.33	97.56	96.26	98.18	65.03	69.83
2	30000	50	98.89	98.6	97.32	97.76	60.23	65.2
3	10000	20	88.62	92.03	79.05	88.61	58.49	65.28
4	10000	20	88.62	92.03	79.05	88.61	58.49	65.28
5	30000	50	98.89	98.6	97.32	97.76	60.23	65.2
6	20000	35	95.17	97.26	91.75	97.49	59.77	64.7
7	10000	35	93.01	95.17	87.25	94.03	69.4	75.64
8	10000	50	97.41	97.82	95.45	97.38	71.11	76.94
9	30000	20	95.76	96.04	95.19	96.52	64.6	65.39
10	10000	35	93.01	95.17	87.25	94.03	69.4	75.64
11	10000	50	97.41	97.82	95.45	97.38	71.11	76.94
12	20000	20	92.19	94.93	87.12	93.95	52.11	57.3
13	20000	50	98.15	99.1	96.38	98.96	58.22	63.03
14	20000	20	92.19	94.93	87.12	93.95	52.11	57.3
15	10000	20	88.62	92.03	79.05	88.61	58.49	65.28
16	20000	35	95.17	97.26	91.75	97.49	59.77	64.7
17	10000	50	97.41	97.82	95.45	97.38	71.11	76.94
18	30000	20	95.76	96.04	95.19	96.52	60.64	65.39
19	20000	50	98.15	99.1	96.38	98.96	58.22	63.03
20	20000	50	98.15	99.1	96.38	98.96	58.22	63.03
21	30000	20	95.76	96.04	95.19	96.52	60.64	65.39
22	20000	20	92.19	94.93	87.12	93.95	52.11	57.3
23	30000	35	97.33	97.56	96.26	98.18	65.03	69.83
24	20000	35	95.17	97.26	91.75	97.49	59.77	64.7
25	10000	35	93.01	95.17	87.25	94.03	69.4	75.64
26	30000	50	98.89	98.6	97.32	97.76	60.23	65.2
27	30000	35	97.23	97.56	96.26	98.18	65.03	69.83

### Analytical Methods

The chemical oxygen demand (Standard code: 5220D), biological oxygen demand (5210 B), TKN (4500 A), total phosphorous (4500-P A), alkalinity (by titration method), and turbidity (by nephelometric method) were performed according to the standard methods. Gravimetric methods were used for the determination of mixed liquor suspended solids (MLSS) and mixed liquor volatile suspended solids (MLVSS) after filtration and drying at temperatures 105 °C and 550 °C, respectively. For COD, a colorimetric method with closed reflux method was developed. The TKN and the BOD were determined by TKN meter (Gerhardt model) and BOD meter, respectively. A spectrophotometer (DR 5000, Hach, Jenway, USA) at 520 nm was used for measuring NH_3_-N and TP. Wastewater DO levels were determined by using a DO probe (Oxi, Germany). The pH, conductivity and the oxidation reduction potential (ORP) were monitored by a pH meter (WTW, Germany).

## Results and Discussion

### Reactor start-up

**Figure 2:**
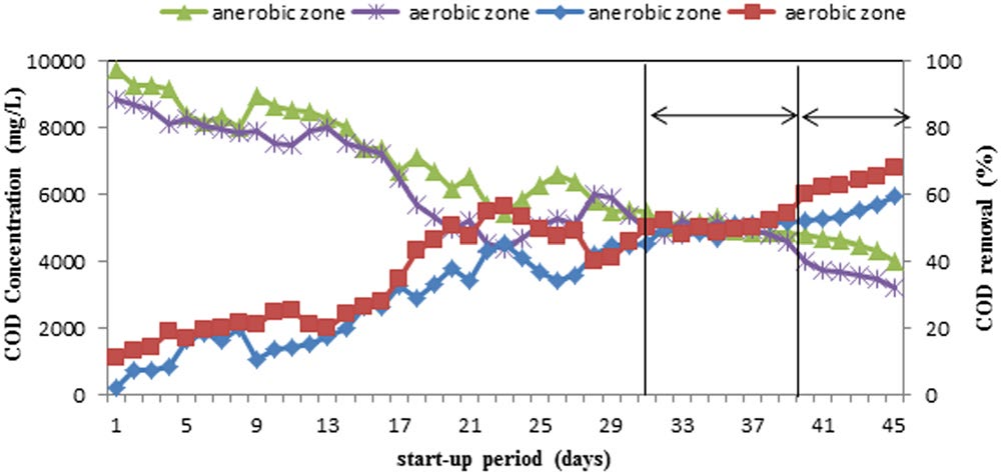
Profile of COD removal during acclimatization of sludge.

The primary analysis of MLL showed that the BOD_5_/COD ratio is higher than 0.7, the samples are highly biodegradable, and therefore the considered landfill is young. At start-up period, acclimatization was completed for 45 days at 20% volumetric GAC, and COD was about 10.000 mg/L. The end-point of start-up was selected by COD removal percentage lower than 5% at the 8th day of operation time (31 to 39 day). At this condition, biological system is reached to a pseudo steady state. These variations can be observed in Fig. 2. Accordingly, the COD removal percentages in anaerobic and aerobic zone were 52% and 54.4%, respectively. The COD declined from 10000 mg/L to 4807 mg/L in anaerobic zone, and to 4561 mg/L in the aerobic zone. An efficiency improvement phase occurred after about 40 to 45 day. This may be related to a system upgrading due to a higher bacterial mass and an accumulation of biodegrading enzymes after the steady state phase. In this improved phase, an opportunity for the production of enzymes via long operation time was provided. At high biodegradable amount of MLL (BOD/COD ~ 0.7) and primary operation time, the cross-link metabolisms occurred successfully. The co-metabolism in the presence of high biodegradable matter can be affected and is completed by operating at the proper contact time. All the required enzymes for biodegradable and resistant compounds in MLL is generated. LaPara *et al*.^20^ demonstrated that the specific activity of catabolic enzyme is increased at the proper HRTs^20^. In steady state phase, the generation of the responsible enzyme for resistant-compounds was improved. Consequently, a high value of removal efficiency for COD is obtained.

### Effect of HRT

**Figure 3:**
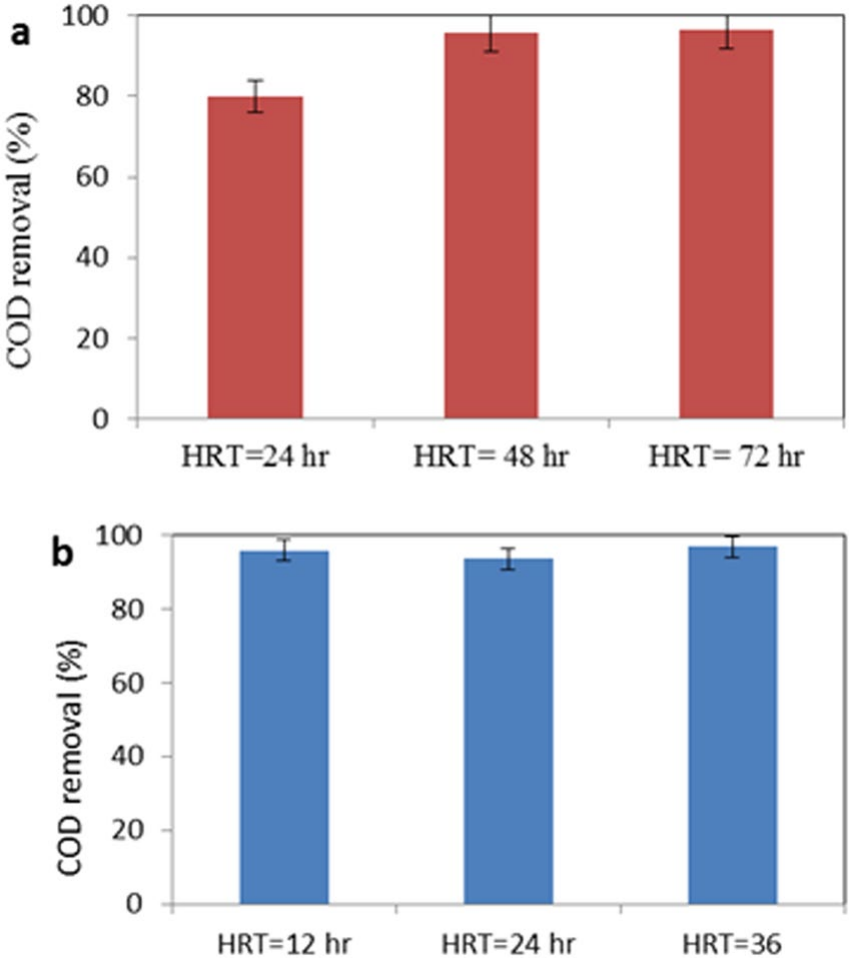
Determination of optimal HRT for anaerobic and aerobic units.

HRT is one of the important variables that can have significant effects on a biological process, and it provides an adequate reaction time between the biomass/biofilm and the substrate material^21^. Therefore, determining an optimum HRT for decreasing the treatment costs, and for increasing the system efficiency at different anaerobic–aerobic units is important. Figure 3a and b shows the effect of different HRT in constant loading rate. At this stage, anaerobic unit was operated at 24, 48 and 72 h, while the aerobic unit was operated at 12, 24 and 36 h. Under anaerobic conditions as a function of the operating time, COD removal was in the range of 79.9 ± 2.7, 95.6 ± 1.5 and 96.5 ± 1.19%, respectively. In the anaerobic stage, because the difference in COD removal values between both the HRT (48 and 72 h) was not significant, the HRT~48 h was chosen as optimum HRT with the original loading. At same HRTs (for example 24 h) and in comparison with high rate (for 0.1–10 kg COD/m^3^/day) anaerobic processes such as Anaerobic Filters (AF), Anaerobic expanded/Fluidized bed rectors, Upflow Anaerobic Sludge Blanket (UASB) reactor, IAnA-BioGACSBR have been reported with notable results (efficiency~80% for 10 kg COD/m^3^. day). In the aerobic zone, the obtained COD removal was about 95.74 ± 1.5, 93.46 ± 1.34 and 96.7 ± 0.9%, respectively. Due to negligible difference between HRTs at 12 h with 24 and 36 h, the HRT equal to 12 h was selected as the optimal point for HRT. It can be found from aerobic results that a considerable amount of COD (95.74%) was degraded in regard to the conventional treatment methods such as activated sludge modification process.

### ANOVA and statistical analysis

**Figure 4:**
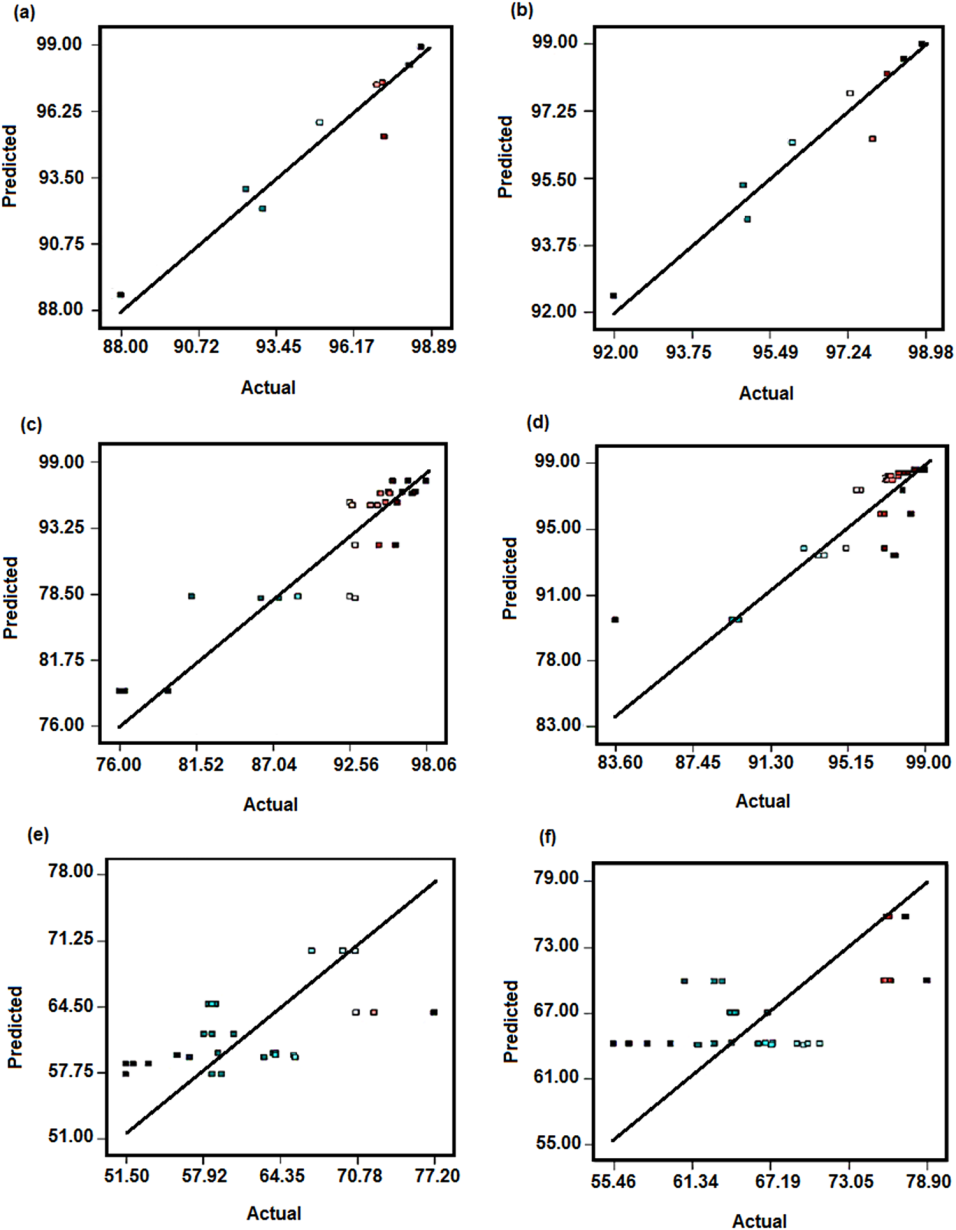
Response surface plot for COD and BOD_5_ removal; anaerobic (**a**,**c**) aerobic (**b**,**d**).

A FFD of twenty seven runs was conducted in the pilot scale to determine the responses. As a function of *x*_*1*_ and *x*_*2*_, a mathematical equation is drawn. Table 5 illustrates ANOVA and statistical parameters that were obtained based on experimental data. In order to specify the accuracy of fit, ANOVA is used. Usually, the adequacy of the model can be evaluated by diagnostic plots, such as a plot of predicted versus actual values. As illustrated in Fig. 4a–f, the predicted values versus actual values are presented. The predicted values were calculated from the final coded mathematical equations.

To investigate the effects of the considered variables on the COD, BOD_5_ and TKN removal efficiency, the dependencies of these responses to the variables were analyzed and modeled. A reduced quadratic and Linear model was selected to describe the variation removal efficiency of the COD, BOD and TKN (Table 6). Table 6 summarizes the statistical parameters of the obtained equations. As presented in Table 6, the main effects of the two factors (*x*_*1*_: loaded COD and *x*_*2*_: GAC filling ratio percentage) are significant model terms. However, the effect of *x*_*2*_ is much more important than that of *x*_*1*_. This effect is more specific for anaerobic process rather than aerobic (see numerical coefficients of *x*_*1*_ and *x*_*2*_). In comparison with aerobic process, variables effectiveness is so close. From a comprehensive overview of the provided models, it can be seen that Linear and quadratic models were fitted with experimental data. The COD removal models for anaerobic and aerobic processes were confirmed with polynomial (quadratic) models. These findings suggest that the COD removal can be affected by many variables. These variables include operation condition, HRT, COD removal mechanisms (anaerobic/aerobic degradation, adsorption, sedimentation etc.), COD loading, filling ratio, COD/TKN ratio, granule maturation. On the other hand, a quadratic equation can be obtained when both biodegradable and resistant compound are removed simultaneously. The COD removal models with high determination coefficient (R^2^ = 0.93 for both anaerobic and aerobic zones) demonstrates that there is a desirable conformity between experimental data and models. As seen in BOD removal models, linear model refers to a strong effective parameter or mechanism that has a predominant effect than others. With regard to high ratio of BOD/COD (~0.7), it can be concluded that possibly the biodegradation is the effective mechanisms by the rapid degradation of organic via aerobic process. Also, the linear model for TKN in aerobic process is related to limitation of process by improper amounts of BOD/TKN ratio (>10). When BOD/TKN ratio >4, it provides limited denitrification and under this condition the nitrification is not affected significantly. However, nitrification byproducts accumulation occurs^22^. At this time, TKN removal is restricted to dissimilation.

**Table 6:** Statistical results and derived equations.

Response	Zone	Modified Equations with significant terms (*x*_*1*_: inlet COD (mg/L) and *x*_*2*_: GAC %)	Model type	R^2^	adequate precision	P-value
removal COD	Anaerobic	95.51 + 2.16 *x*_*1*_ + 2.98 *x*_*2*_ − 1.41 *x*_*1*_ *x*_*2*_ − 1.44 *x*_*1*_^2^ − 0.57 *x*_*2*_^2^	*Quadratic*	0.93	28.787	<0.0001
	Aerobic	97.26 + 1.20 *x*_*1*_ + 2.09 *x*_*2*_ − 0.8 *x*_*1*_ *x*_*2*_ − 0.90 *x*_*1*_^2^ − 0.25 *x*_*2*_^2^	*Quadratic*	0.93	28.669	<0.0001
removal	Anaerobic	91.75 + 4.50 *x*_*1*_ + 4.63 *x*_*2*_ − 3.57 *x*_*1*_ *x*_*2*_	*Linear*	0.84	17.366	<0.0001
	Aerobic	96.80 + 2.07 *x*_*1*_ + 2.50 *x*_*2*_ − 1.88 *x*_*1*_ *x*_*2*_ − 1.39 *x*_*1*_^2^ − 0.37 *x*_*2*_^2^	*Quadratic*	0.74	12.606	<0.0001
removal	Anaerobic	59.77 − 2.18 *x*_*1*_ + 3.05 *x*_*2*_ − 3.26 *x*_*1*_ *x*_*2*_ + 7.45 *x*_*1*_^2^ − 4.60 *x*_*2*_^2^	*Quadratic*	0.73	10.564	0.0242
	Aerobic	67.03 − 2.91 *x*_*1*_ + 2.68 *x*_*2*_ − 2.96 *x*_*1*_ *x*_*2*_	*Linear*	0.36	5.508	0.0006

**Figure 5:**
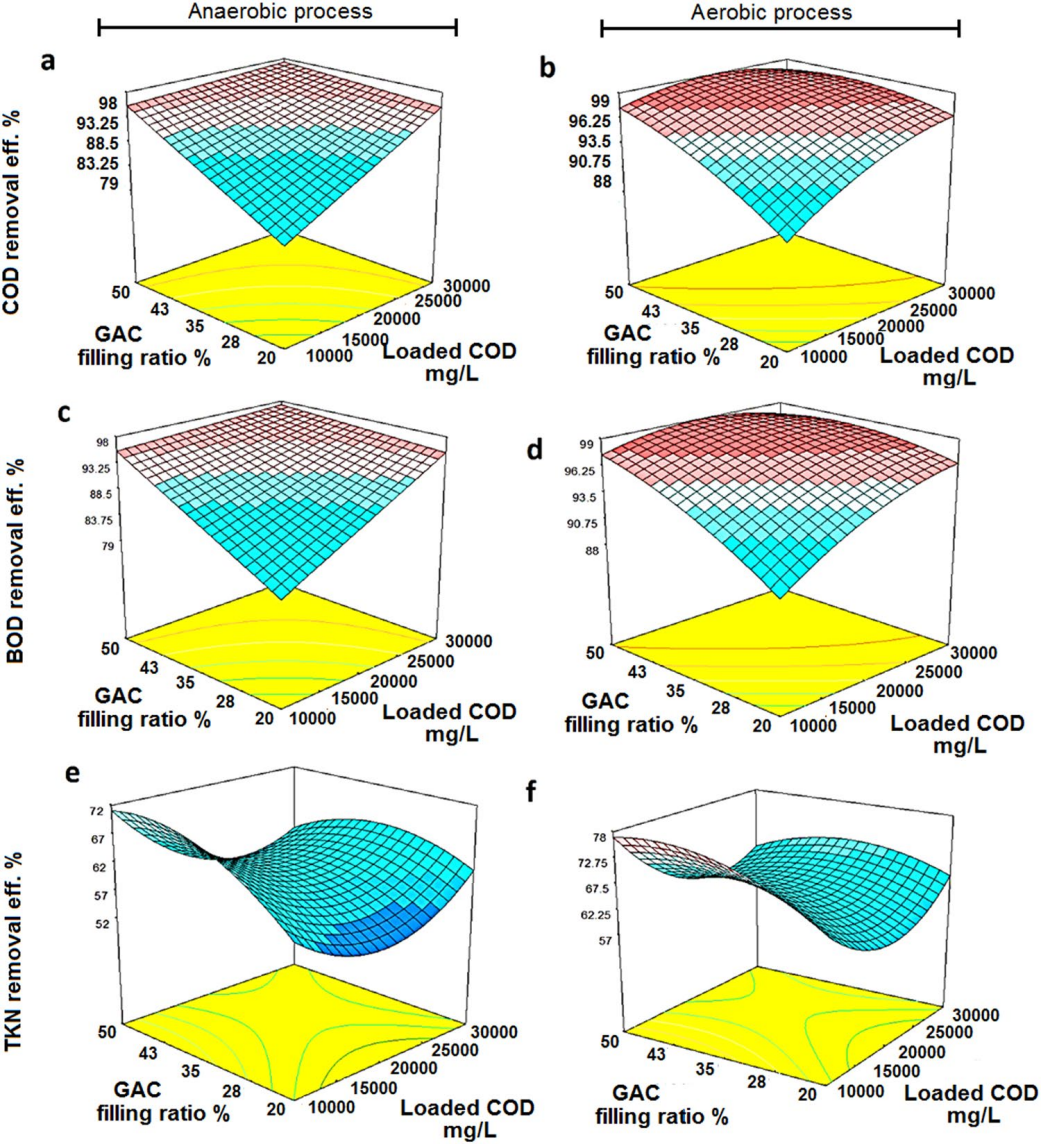
Predicted vs. actual values plot for COD, BOD and TKN removal; anaerobic (**a**,**c** and **e**) aerobic (**b**,**d** and **f**).

Figure 5 illustrates the variation of responses in three dimension plots. According to Fig. 5a and b, the response linearly increased with an increase in the COD concentration and filling ratio percentage [COD removal in anaerobic zone (98.9%) and in aerobic zone (99%)]. In anaerobic and aerobic zone, the maximum values of BOD removal were obtained, 98.54% and 98%, at the highest values of the independent factors (30000 mg/L and 50%), respectively (Fig. 5c and d). Similar pattern for COD and BOD_5_ is seen in anaerobic as well as aerobic processes. This reveals that the MLL was easily degradable (BOD_5_/COD 0.7) and anaerobic process successfully removed the higher amount of loaded organic matter. As mentioned, the effect of *x*_2_ (GAC filling ratio percentage) was more effective than loaded COD concentration in the anaerobic zone. These results suggest that the efficiency of anaerobic process increases with higher filling ratio when the system is operated under high rate condition. Significant efficiency for all responses can be related to the high surface area to volume ratio and porosity. As a result, large adsorption capacity of GAC could provide high substrate removal efficiency with high process stability^23^. Loukidou and Zouboulis reported that the use of GAC moving-bed biofilm SBR process was capable to remove the most biodegradable organic carbon, together with the major fraction of COD^2^. Anaerobic SBR has been studied by Timur *et al*.^24^ for young landfill leachate and they reported COD removal rates of about 64–85%^24^. To treat the textile dyestuff, an anaerobic–aerobic SBR was used and the significant COD removal (higher than 85%) were obtained for a COD loading of 500 mg/L^25^. Aziz *et al*.^15^ proposed that a powdered activated carbon (PAC)-SBR was able to treat the landfill leachate, and the PAC-SBR displayed superior performance in terms of removal efficiencies compared to single SBR^15^. Under optimal condition including aeration rate of 1 L/min and contact time of 5.5 h, the PAC-SBR provided about 64.1% of COD removal^15^. As seen from Fig. 5e and f, the increase in filling ratio percentage caused an increase in the removal efficiency for both anaerobic and aerobic processes. The maximum TKN removal of 77.2% and 78.9% were obtained at COD ~ 10.000 mg/L with a filling ratio of about 35%. Because of the high BOD/TKN ratios (4.7 to 12), it was assumed that nitrifiers were not significantly cultured in the SBR reactor^22^. It is evident that the nitrogen is likely consumed as a nutrient for the synthesis of new cells via assimilation pathway^26^. This result suggested that the TKN removal efficiency can also be completed by adsorption into GAC surface and porous area, and also transferred to nitrification byproducts and their accumulations. According to Fig. 5e and f, it can be asserted that the TKN removal rate declined primarily due to the negative interaction between nitrification and heterotrophic bacteria in both anaerobic as well as aerobic units. This negative effect occurs when the carbon to nitrogen ratio (C/N), is improper, and consequently, a small population of nitrogen oxidizing bacteria (such as nitrite oxidizing bacteria) can be active so that nitrite and other nitrification byproducts are accumulated^21^. After the initial drop of TKN removal efficiency, an ascending removal rate was observed, which could be due to the adequate acclimatization of nitrifying and denitrifying bacteria in the connection with higher COD loading rates. In a similar study reported by Sirianuntapiboon *et al*.^27^, they demonstrated that TKN and ammonium in a GAC-SBR system decreased the nitrite accumulation^27^. The TKN removal rather than COD and BOD removal efficiency via higher filling ratio of GAC was increased gradually, and the COD and BOD removal efficiency was improved rapidly. Similar results reported by Sirianuntapiboon *et al*.^27^ showed that organic matter and dye could be removed rapidly with higher efficiency^27^. They proposed that this fact has been associated with the growth of biosludge (growth association mechanism)^28^.

### Optimization

**Figure 6:**
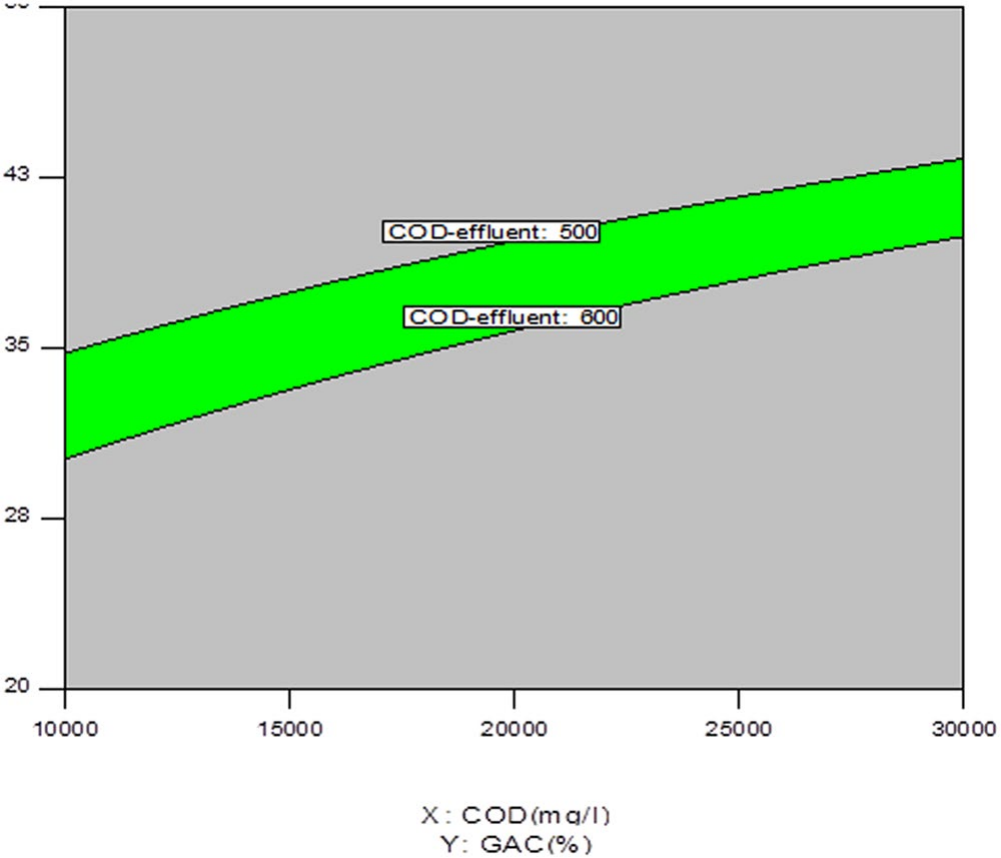
Effluent optimization for effluent discharge to municipal wastewater collection network.

Table 7 presents the option criteria for discharging MLL effluent to municipal wastewater and the corresponding values of the independent variables. In this regard, the quality of MLL effluent has to comply with increasing stringent discharge standard. It is clear that the performance of the high rate IAnA-BioGACSBR can be used for discharging MLL effluent to municipal wastewater network. The optimum zone was obtained based on responses of COD in the range 500–600 mg/L and BOD in the range 200–300 mg/L. Figure 6 illustrates the achieved optimum zone.

**Table 7:** Solution runs for MLL effluent discharge to municipal wastewater.

Number of Solutions	Initial COD (mg/l)	Filling ratio (%)	Effluent-COD (mg/l)	Effluent-BOD (mg/l)
1	13266	34	552.025	290.051
2	22546	38	572.292	284.027
3	27002	41	527.439	262.341
4	16810	38	513.808	263.954
5	15900	34	581.81	298.829
6	11726	34	536.569	286.03
7	13394	36	523.421	275.067
8	14848	34	571.936	296.316
9	17100	38	502.552	258.1
10	18858	37	567.247	286.337
11	12316	33	565.257	299.447
12	23322	39	543.306	270.59
13	21691	40	500.05	252.45
14	28828	40	591.687	288.401
15	28754	42	522.261	260.183
16	28202	40	576.99	282.575
17	18762	36	585.789	295.198
18	13352	35	545.169	286.319
19	12329	34	549.72	291.298
20	17424	36	558.166	284.36
21	14044	35	554.779	289.562
22	11866	33	553.191	294.4
23	19002	36	591.11	297.29
24	19276	39	510.806	259.397
25	24974	38	592.468	290.71
26	11916	34	530.04	282.084
27	18430	38	524.062	266.61
28	18778	36	582.241	293.504
29	21916	38	562.2	280.154
30	18042	38	510.742	260.793

### Effect of GAC addition

**Figure 7:**
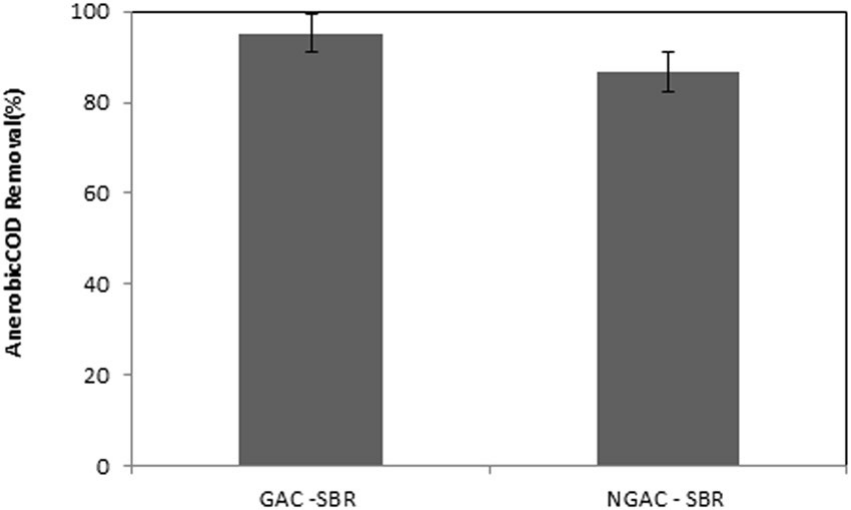
COD removal efficiencies of GAC- ASBR and NGAC –ASBR.

To evaluate the effect of GAC-SBR versus SBR, this phase of experiment under same condition was conducted. The results obtained for the test of GAC addition are shown in Fig. 7. Accordingly, the COD removal efficiencies of two systems were analyzed and it was found that high rate IAnA-BioGACSBR has clearly significant effect on COD biodegradation as compared to others. The average efficiencies of COD removal from MLL in the SBR and the GAC-SBR were determined at about 95.17% and 86.68% (with p-value < 0.001), respectively.

## Conclusions

The treatability of MLL was studied by using a novel IAnA-BioGACSBR. Under anaerobic and aerobic conditions HRTs (48 and 12 h) were selected as optimum HRT with highest COD removal as 95.6 ± 1.5%, 95.74 ± 1.5, respectively. In order to model and optimize this SBR, experiments were conducted based on FFD and RSM. The results showed that the maximum BOD removal obtained was about 98.9 and 99% when the COD is 30.000 mg/L and the filling ratio is 50%. The maximum COD removal percentages were found over 98% (at COD ~ 10000 mg/L and GAC of 35%). Under these conditions the highest removal of TKN was 77.2% and 78.9%, respectively. Under optimum zone, the MLL can be appropriately discharged into municipal wastewater.
